# Identification of BIRC6 as a novel intervention target for neuroblastoma therapy

**DOI:** 10.1186/1471-2407-12-285

**Published:** 2012-07-12

**Authors:** Fieke Lamers, Linda Schild, Jan Koster, Frank Speleman, Ingrid Øra, Ellen M Westerhout, Peter van Sluis, Rogier Versteeg, Huib N Caron, Jan J Molenaar

**Affiliations:** 1 Department of Oncogenomics, Academic Medical Center, University of Amsterdam, Meibergdreef 15, PO box 22700, Amsterdam, AZ 1105, The Netherlands; 2 Center for Medical Genetics, Ghent University Hospital, Ghent, Belgium; 3 Department of Pediatric Oncology, Skåne University Hospital, Lund University, Lund, Sweden; 4 Department of Pediatric Oncology, Emma Kinderziekenhuis, Academic Medical Center, University of Amsterdam, Amsterdam, The Netherlands; 5 Department of Oncogenomics, M1-132, Academic Medical Center, University of Amsterdam, Meibergdreef 9, Amsterdam, AZ 1105, The Netherlands

**Keywords:** Neuroblastoma, BIRC6, DIABLO, Apoptosis, Cancer

## Abstract

**Background:**

Neuroblastoma are pediatric tumors of the sympathetic nervous system with a poor prognosis. Apoptosis is often deregulated in cancer cells, but only a few defects in apoptotic routes have been identified in neuroblastoma.

**Methods:**

Here we investigated genomic aberrations affecting genes of the intrinsic apoptotic pathway in neuroblastoma. We analyzed DNA profiling data (CGH and SNP arrays) and mRNA expression data of 31 genes of the intrinsic apoptotic pathway in a dataset of 88 neuroblastoma tumors using the R2 bioinformatic platform ( http://r2.amc.nl). BIRC6 was selected for further analysis as a tumor driving gene. Knockdown experiments were performed using BIRC6 lentiviral shRNA and phenotype responses were analyzed by Western blot and MTT-assays. In addition, DIABLO levels and interactions were investigated with immunofluorescence and co-immunoprecipitation.

**Results:**

We observed frequent gain of the *BIRC6* gene on chromosome 2, which resulted in increased mRNA expression. BIRC6 is an inhibitor of apoptosis protein (IAP), that can bind and degrade the cytoplasmic fraction of the pro-apoptotic protein DIABLO. *DIABLO* mRNA expression was exceptionally high in neuroblastoma but the protein was only detected in the mitochondria. Upon silencing of BIRC6 by shRNA, DIABLO protein levels increased and cells went into apoptosis. Co-immunoprecipitation confirmed direct interaction between DIABLO and BIRC6 in neuroblastoma cell lines.

**Conclusion:**

Our findings indicate that BIRC6 may have a potential oncogenic role in neuroblastoma by inactivating cytoplasmic DIABLO. BIRC6 inhibition may therefore provide a means for therapeutic intervention in neuroblastoma.

## Background

BIRC6 (also known as BRUCE or APOLLON) is a cytoplasmic protein with a dual role. Firstly, BIRC6 has an anti-apoptotic function in the intrinsic apoptotic pathway by antagonizing the pro-apoptotic DIABLO protein. BIRC6 can bind the cytoplasmic DIABLO fraction and induce ubiquitination and proteasomal degradation of this protein [1,2]. BIRC6 thereby protects against the pro-apoptotic function of DIABLO. DIABLO is a mitochondrial protein which is released into the cytoplasm upon an apoptotic stimulus. This release is regulated by the levels of the BH3 family proteins, which induce pore formation in the mitochondrial membrane [3-5]. Cytoplasmic DIABLO can bind to the BIR domains of BIRC2 (cIAP1), BIRC3 (cIAP2) and BIRC4 (XIAP), thereby inhibiting the anti-apoptotic function of these proteins [6]. A second function of BIRC6 has been shown in recent studies where BIRC6 was required for abscission and membrane delivery during the midbody ring formation during cell division [7,8].

*BIRC6* is highly expressed in several types of cancer. *BIRC6* over-expression in acute myeloid leukemia is correlated with a poor outcome [9]. A genome wide screening of chromosomal aberrations in Burkitt’s lymphoma showed that a region of 2p including the *BIRC6* gene was gained in a few samples [10]. Additionally, high *BIRC6* expression in colon cancer stem cells is associated with drug resistance [11].

Neuroblastoma are pediatric tumors that originate from the embryonal precursor cells of the sympathetic nervous system. High stage tumors have a poor prognosis with 20 to 40% overall survival [12-14]. *BIRC6* is located on chromosome 2p in the region which shows frequent gain in neuroblastoma [12]. This region includes both *MYCN* and *ALK*, two well characterized oncogenes in neuroblastoma. Amplification of *MYCN* occurs in 20–30% of neuroblastoma and strongly correlates with a poor prognosis [12-15]. *ALK* was recently found to be mutated in 6-10% of primary neuroblastoma [16-20]. *MYCN* amplification and *ALK* mutations seem independent of the gain of chromosome 2p [21] and therefore other additional tumor driving genes could be located on this frequently gained region.

The apoptotic pathway has been widely investigated in neuroblastoma and only a few tumor driving events have been described. *TP53* is mostly intact in primary neuroblastoma although functional defects in the p53 pathway have been described [22]. *Caspase 8 (CASP8)* is hypermethylated and thereby inactivated in some neuroblastoma resulting in an inactive extrinsic apoptotic pathway [23]. The IAP *BIRC5 (Survivin)* is located on the chromosome 17q region which is frequently gained in neuroblastoma and high BIRC5 expression correlates with a poor prognosis [24-26]. Also, the anti-apoptotic mitochondrial *BCL2* protein is highly expressed in neuroblastoma. Targeted inhibitors against BIRC5 [27-32] and BCL2 [33] are currently being tested for clinical implementation, however, the poor prognosis of high grade neuroblastoma makes the identification of additional targets for therapeutic intervention desirable.

To identify patterns in aberrations of genes involved in intrinsic apoptotic signaling we combined high throughput analysis of DNA copy number and mRNA expression of these genes in a dataset of 88 neuroblastoma tumors. We found *BIRC5* and *BIRC6* to be frequently gained and *CASP9* often lost. Since *BIRC6* was not previously evaluated in a neuroblastoma model, we studied the potency of BIRC6 as a potential new target for neuroblastoma therapy. Silencing of BIRC6 induced apoptosis and up-regulation of DIABLO. We established BIRC6 to physically interact with DIABLO, indicating that BIRC6 can degrade DIABLO very effectively.

## Methods

### Patient samples

We used a neuroblastoma tumor panel for Affymetrix Microarray analysis containing 88 primary neuroblastoma tumor samples of untreated patients of which 87 neuroblastoma tumor samples were also used for CGH analysis and SNP array [34]. All neuroblastoma samples were residual material obtained during surgery for diagnostic purposes and immediately frozen in liquid nitrogen. Ethical approval of the Dutch Medical Ethical Committee was not needed for the use of surplus materials. However, informed consent was taken from the parents of the patients for use of this material which is archived at the Academic Medical Center from the University of Amsterdam. The data were deposited in the NCBI Gene Expression Omnibus ( http://www.ncbi.nlm.nih.gov/geo/) under accession number GSE16476 [35]. Public available neuroblastoma datasets we used were of Delattre [36] and Lastowska (geo ID: gse13136). Public available datasets were used for comparing neuroblastoma with normal tissues (Roth dataset, geo ID: gse3526) and adult tumors (EXPO dataset, geo ID: gse2109).

### Affymetrix mRNA expression analysis

Total RNA of neuroblastoma tumors was extracted using Trizol reagent (Invitrogen, Carlsbad, CA) according to the manufacturer’s protocol. RNA concentration and quality were determined using the RNA 6000 nano assay on the Agilent 2100 Bioanalyzer (Agilent Technologies). Fragmentation of cRNA, hybridization to hg-u133 plus 2.0 microarrays and scanning were performed according to the manufacturer’s protocol (Affymetrix inc).

### Array CGH analysis

High-molecular-weight DNA was isolated from tumor tissue by a standard salt-chloroform extraction method [37]. For reference DNA we obtained healthy tissue. We used a custom 44 K Agilent aCGH chip, enriched for critical regions of loss/gain for neuroblastoma (10 kb resolution), miRNAs/T-UCRs (5 oligos/gene) and cancer gene census genes (5 oligos/gene) (Agilent Technologies). A total of 150 ng of tumor and reference DNA was labeled with Cy3 and Cy5, respectively (BioPrime ArrayCGH Genomic Labeling System, Invitrogen). Further processing was done according to the manufacturer’s guidelines. Features were extracted using the feature extraction v10.1.0.0.0 software program. Data were further analyzed using the R2 web application (see below). Circular binary segmentation was used for scoring the regions of gain, amplification and deletion.

### Whole-genome genotyping

Tumor DNA was extracted as previously described, quantified with NanoDrop and the quality was determined by the Abs 260/280 and 230/260 ratio. SNP arrays were processed for analysis of copy number variations with the Infinium II assay on Human370/660-quad arrays containing > 370 000/> 660 000 markers and run on the Illumina Beadstation (Swegene Centre for Integrative Biology, Lund University – SCIBLU, Sweden) according to the manufacturer’s recommendations. Raw data were processed using Illumina’s BeadStudio software suite (Genotyping module 3.0), producing report files containing normalized intensity data and SNP genotypes. Subsequently, log 2 Ratio and B-allele frequency data were imported into the R2 web application for detailed analysis and comparison with the CGH and expression data.

### Bioinformatics

All data were analyzed using the R2 web application, which is publicly available at http://r2.amc.nl. The expression data were normalized with the MAS5.0 algorithm within the GCOS program of Affymetrix Inc. Target intensity was set to 100. For scoring genomic aberrations of the 31 included genes (see below and Table 1), we considered CGH aberrant if the logfold value was more than 0.45 for gain or less than −0.45 for loss and if a breaking point was clearly visible. We excluded whole chromosome gains or losses. Also, the detected gains or losses had to be confirmed by SNP array.

**Table 1:** Selected genes in the intrinsic apoptotic pathway

**Gene family or category**	**Gene (alias)**
Anti-apoptotic members of BCL2 family	BCL2
	MCL1
	BCL2L1 (BCLXL)
	BCL2L2 (BCLW)
	BCL2A1
	BCL2L10
Pro-apoptotic members of BCL2 family	BBC3 (PUMA)
	BCL2L11 (BIM)
	BID
	PMAIP1 (NOXA)
	BAD
	BIK
	HRK
	BCL2L14
	BMF
Mitochondrial permeabilization	BAX
	BAK1
Cytoplasmic pro-apoptotic genes	DIABLO
	CYCS
	APAF1
Caspases, activated by mitochondrial apoptotic pathway	CASP3
	CASP6
	CASP7
	CASP9
IAPs	NAIP
	XIAP
	BIRC5
	BIRC6
	BIRC7
Activation of mitochondrial apoptotic pathway	TP53
	CASP2

### Cell lines

All cell lines were grown in Dulbecco Modified Eagle Medium (DMEM), supplemented with 10% fetal calf serum, 10 mM L-glutamine, 10 U/ml penicillin/streptomycin, Non Essential Amino Acids (1x) and 10 μg/ml streptomycin. Cells were maintained at 37°C under 5% CO_2_. For primary references of these cell lines, see Molenaar et al. [35].

### Lentiviral shRNA production and transduction

Lentiviral particles were produced in HEK293T cells by co-transfection of lentiviral vector containing the short hairpin RNA (shRNA) with lentiviral packaging plasmids pMD2G, pRRE and pRSV/REV using FuGene HD. Supernatant of the HEK293T cells was harvested at 48 and 72 h after transfection, which was purified by filtration and ultracentrifugation. The concentration was determined by a p24 ELISA. Cells were plated at a 10% confluence. After 24 h cells were transduced with lentiviral DIABLO shRNA (Sigma, ‘E8’: TRCN0000004511 and ‘E9’: TRCN0000004512) or BIRC6 shRNA (Sigma, ‘C7’: TRCN0000004157 and ‘C11’: TRCN0000004161) in various concentrations (Multiplicity of infection (MOI): 1–3). SHC-002 shRNA (non-targeting shRNA: CAACAAGATGAAGAGCACCAA) was used as a negative control. Medium was refreshed 24 h after transduction and puromycin was added to select for transduced cells. Protein was harvested 72 h after transduction and analyzed by Western blot.

### Compounds

ABT263, a small molecule BCL2 inhibitor, was dissolved in DMSO to a concentration of 20 mM as stock solution. A final concentration of 200 nM ABT263 was used. For the experiment using this compound we chose SJNB12 instead of IMR32 or SKNSH for its high expression of BCL2 and its subsequent high sensitivity to this compound [33].

Z-Val-Asp(OMe)-Val-Ala-Asp(OMe)-FMK (ZVDVAD-FMK, a widely used CASP2 inhibitor; R&D systems) was added to the cells following manufacturer’s protocol in a concentration of 20 μM.

### Western blotting

Attached and floating cells were harvested on ice 72 h after transduction with shRNA. Cells were lyzed with Laemmlibuffer (20% glycerol, 4% SDS, 100 mM Tris HCl pH 6.8 in mQ). Protein concentrations were quantified with RC-DC protein assay (Bio-Rad, Hercules, USA). Lysates were separated on a 10% SDS-Page gel and transferred onto a blotting membrane (Millipore, IPFL00010). Blocking and incubation were performed in OBB according to manufacturer’s protocol (LI-COR). Primary antibodies used were anti-BIRC6 (Abcam, ab19609), anti-DIABLO (Abcam, ab32023), anti-PARP (Cell Signaling: 9542) and anti-BCL2 (Cell Signalling; 2872). Protein loading was checked by anti-β-actin (Abcam, ab6276) or anti-α-tubulin (Sigma, T5168). The secondary antibodies used were provided by LI-COR. Proteins were visualized with the Odyssey bioanalyzer (LI-COR).

### In cell western

Cells were fixed with 4% paraformaldehyde for 20 min 48 h after transduction with BIRC6 shRNA. Blocking and incubation were performed in OBB according to manufacturer’s protocol (LI-COR). Primary antibodies used were anti-BIRC6 (BD Biosciences, 611193) and anti-β-actin mouse monoclonal (Abcam, ab6276). Proteins were visualized with the Odyssey bioanalyzer (LI-COR) and quantified and corrected for β-actin using the Odyssey software.

### Immunofluorescence

Cells were grown on glass slides in 6-well plates and were fixed with 4% paraformaldehyde in PBS 48 h after transduction. We used anti-DIABLO (Abcam, ab32023) as a primary antibody, and anti-rabbit (Alexa, 11012) as a secondary antibody. Mitochondria were stained using Mitotracker (Invitrogen, M22426). Antibodies were dissolved in 5% ELK in PBS/0.2% tween-20. Slides were stained with DAPI (1:1000) in vectashield (Vector Laboratories).

### Cell fractionation

Protein was harvested and fractionated using the Subcellular Proteome Extraction Kit according to manufacturer’s protocol (Novagen, 539790). Fraction I (cytosol) and II (membrane/organelle) were used for Western blot.

### Co-immunoprecipitation

Cells were lyzed in a buffer containing 150 mM NaCl, 50 mM Hepes, 5 mM EDTA, 0.3% NP-40, 10 mM β-glycerophosphate, 6% glycerol, protease inhibitors (Complete mini, Roche) and Phosphatase inhibitors (5 mM NaF, 1 mM Na2VO3). The antibody used for IP was anti-BIRC6 (Abcam, ab19609); negative controls were anti-flag (Cell Signaling, 2368) and protein without antibody. Protein-G agarose beads (Roche) and antibody were incubated for pre-coupling overnight after which lysates were added and incubated overnight. Immunocomplexes were washed, heated at 95°C for 10 min and loaded on a gel for Western blot. Primary antibodies used were anti-BIRC6 and anti-DIABLO (Abcam; ab32023). Blots were incubated overnight with primary antibodies, after which a one hour incubation step with anti-rabbit IgG (Cell Signaling; 3678) was performed followed by incubation with the secondary antibody provided by LI-COR.

### MTT-assay

Cells were seeded at 30% confluence in a 48-well plate, transduced with BIRC6 shRNA and after 24 h treated with ZVDVAD. Seventy-two hours after treatment, 25 μl of Thiazolyl blue tetrazolium bromide (MTT, Sigma M2128) was added. After 4–6 h of incubation, 250 μl of 10% SDS, 0.01 M HCl was added to stop the reaction. The absorbance was measured at 570 nm and 720 nm using a plate-reader (Biotek).

## Results

### Gain of BIRC5 and BIRC6 and loss of CASP9 in neuroblastoma tumors

**Figure 1:**
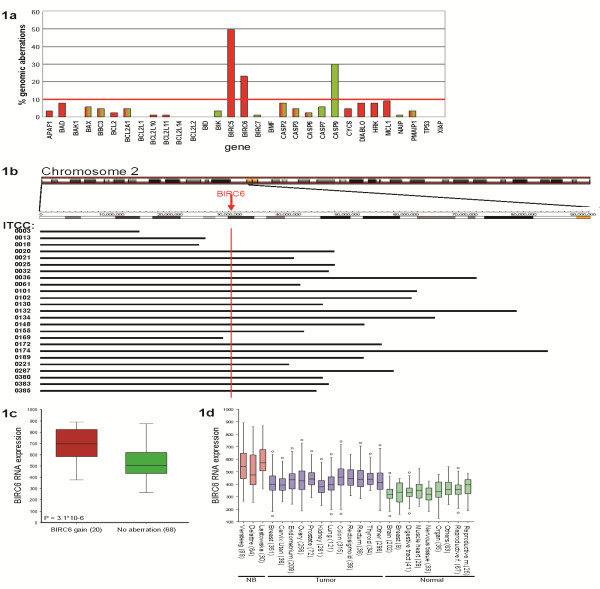
**Gain of BIRC5 and BIRC6 and loss of CASP9 in neuroblastoma tumors. a**: The percentage of genomic aberrations is presented on the Y-axis and all selected genes in the intrinsic apoptotic pathway on the X-axis. Red bars indicate gained genes and green bars indicate lost genes. When a bar is green/red combined, it means that both gains and losses in that gene occurred. The red horizontal line represents the cut-off for further analysis. **b**: Chromosome 2 is represented with the regions of 2p that are gained in our dataset of 88 neuroblastoma tumors. The BIRC6 locus is indicated with a red arrow. **c**: Boxplots of BIRC6 mRNA expression in tumors with or without gain of BIRC6. **d**: Boxplots of BIRC6 mRNA expression in 3 neuroblastoma datasets (red), adult tumors (blue) and various normal tissues (green). The boxes represent the 25^th^ to 75^th^ percentile with the median depicted as a horizontal line. Extremes are indicated by the whiskers, and the presence of outliers is indicated by (o).

To identify patterns in the aberrations of genes involved in intrinsic apoptotic signaling, we combined high throughput analysis of DNA copy number and mRNA expression of these genes. We included all 31 genes that are directly involved in the mitochondrial apoptotic pathway and their downstream target genes (Table 1). Array CGH data of 87 primary neuroblastoma tumor samples were analyzed for copy number variations of the 31 genes included in our intrinsic apoptotic gene panel. Binary segmentation data was used to score the DNA copy number variations and they were subsequently confirmed using log fold data from SNP array analyses of the same tumors. Only three genes show DNA copy number aberrations at a frequency above 10% (Figure 1a). The *BIRC5* gene, which is located in the smallest region of overlap (SRO) of gain of chromosomal band 17q25 [12], is gained in 49% of the tumors. *CASP9* is located at the SRO of deletions of 1p36 [12] and it is lost in 30% of the tumors. *BIRC6*, which is located on 2p22, is gained in 24% of the neuroblastoma tumors. Distal chromosome 2p is a known region of gain in neuroblastoma. *BIRC6* is not located in the SRO of this gained region, but is gained in 84% of the tumors with 2p gain (Figure 1b). Of these three genes, *BIRC6* was not studied before in neuroblastoma. We therefore investigated whether the gain of *BIRC6* resulted in aberrant expression. We compared *BIRC6* expression in tumors with and without *BIRC6* gain, which shows that tumors with *BIRC6* gain have significantly higher *BIRC6* RNA levels (Student *T*-test: P = 3.1*10^-6^) (Figure 1c). Moreover, *BIRC6* is also highly expressed compared to several adult tumors and various normal tissues (Figure 1d). These findings suggest that the aberrant expression of *BIRC6* is at least partially caused by genomic aberrations often occurring in neuroblastoma tumors.

### BIRC6 knockdown induces apoptosis in neuroblastoma cells

**Figure 2:**
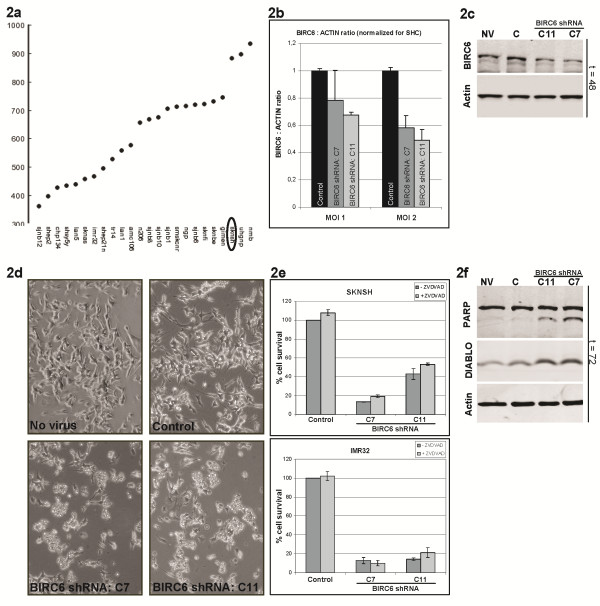
**Knockdown of BIRC6 in SKNSH induces apoptosis. a**: BIRC6 mRNA expression in 24 neuroblastoma cell lines. **b**: In cell western of SKNSH 48 h after transduction. The Y-axis represents the ratio between BIRC6 and Actin protein expression as determined by the Odyssey bioanalyzer. The X-axis represents the concentration BIRC6 shRNA that was added. Black bars are cells transduced with control virus (SHC002), dark grey: C7 BIRC6 shRNA and light grey: C11 BIRC6 shRNA. MOI = Multiplicity of Infection. 2**c**: Western blot of SKNSH 48 h after transduction with no virus (NV), control virus SHC002 (C) or BIRC6 shRNA (C11 and C7). Blots were incubated with BIRC6 and actin antibodies. **d**: Pictures were made 72 h after transduction before protein harvest with a 100x magnitude. **e**: MTT-assay of SKNSH and IMR32 transduced with control virus (SHC) or BIRC6 shRNA (C7 and C11). The dark grey bars represent cells transduced with virus alone; the light grey bars represent cells that are treated with BIRC6 shRNA combined with ZVDVAD, a CASP2 inhibitor. **f**: Western blot of SKNSH 72 h after transduction with no virus (NV), control virus SHC002 (C) or BIRC6 shRNA (C11 and C7). Blots were incubated with PARP, DIABLO and actin antibodies.

We investigated whether the high *BIRC6* levels indeed counteract apoptosis in neuroblastoma. We used 2 lentiviral shRNAs targeting different parts of the coding sequence of *BIRC6*. SKNSH, one of the neuroblastoma cell lines with the highest BIRC6 expression, was transduced with these vectors (Figure 2a). BIRC6 is a 528 kD protein and difficult to assess with Western blot. Therefore we first analyzed BIRC6 protein levels by in cell Western. For this method cells are fixed directly in the culture well and stained with a BIRC6 antibody. Analysis shows concentration dependent down-regulation of BIRC6 protein with both BIRC6 shRNAs at 48 h after transduction (Figure 2b). Although less optimal, we can confirm BIRC6 down-regulation using Western blot (Figure 2c). We then investigated whether BIRC6 silencing induced apoptosis. Light microscopy shows a decreased cell number and cell death at 72 h after BIRC6 silencing (Figure 2d). This is confirmed by MTT assays, which shows strongly reduced cell viability after transduction with both BIRC6 shRNAs (Figure 2e, dark grey bars). This phenotype is caused by an apoptotic response demonstrated by PARP cleavage at 72 h after transduction with both BIRC6 shRNAs on western blot (Figure 2f). These findings confirm an anti-apoptotic role for BIRC6 in neuroblastoma cells.

### High BIRC6 levels keep cytoplasmic DIABLO levels low

The BIRC6 protein functions both by silencing of DIABLO and in the formation of the midbody ring during cell division. Inhibition of each of these functions can result in cell death. We therefore investigated which process causes apoptosis after BIRC6 silencing in neuroblastoma. Apoptosis induced after inhibiting the midbody-related function of BIRC6 has been found to be mediated by CASP2 [7,8,38]. We inhibited CASP2 by ZVDVAD, a widely used CASP2 inhibitor [39-41] that we have previously used to show that apoptosis induced by silencing of BIRC5 is mediated by CASP2 [26]. ZVDVAD however does not inhibit apoptosis induced by BIRC6 knockdown in SKNSH and IMR32 cells (Figure 2e). This indicates that this process is not CASP2-mediated, implying that the role of BIRC6 in neuroblastoma is not essential for completion of cell division during midbody ring formation.

**Figure 3:**
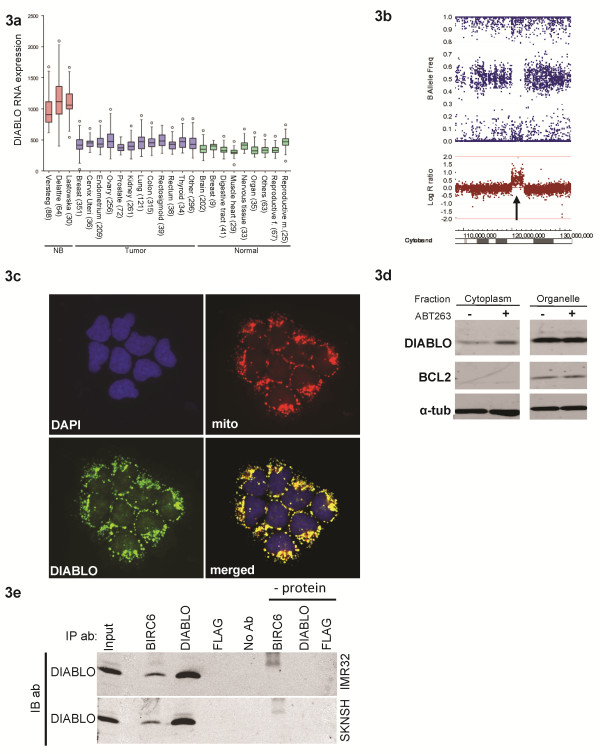
**DIABLO mRNA and protein expression. **3**a**: Boxplots of DIABLO mRNA expression in 3 neuroblastoma datasets (red), adult tumors (blue) and various normal tissues (green). The boxes represent the 25^th^ to 75^th^ percentile with the median depicted as a horizontal line. Extremes are indicated by the whiskers, and the presence of outliers is indicated by (o). 3**b**: Array CGH of NGP of the region of chromosome 12q24.31 in which DIABLO is located (arrow). Both the B allele frequency (top) and the Log R ratio (bottom) are shown. The chromosome region is shown underneath the picture. 3**c**: Immunofluorescence of untreated IMR32 cells. Blue is DAPI, red is mitotracker, green is DIABLO antibody. In the right lower corner the merged pictures are shown. 3**d**: Cell fractionation of SJNB12 cells 24 h after addition of ABT263. The cytoplasmic fraction (cyto) and organelle fraction (organelle) are shown. Blots were incubated with DIABLO, BCL2 and a-tubulin antibodies. 3**e**: Co-immunoprecipitation of IMR32 (top) and SKNSH (bottom) with BIRC6 and DIABLO antibodies. Negative control was the immunoprecipitation antibody Flag. Also a protein sample without antibody and for every antibody a sample without protein was used as negative control. Both blots were incubated with DIABLO antibody. IP antibodies are indicated above the blots.

Alternatively, BIRC6 functions as an IAP that binds DIABLO in the cytoplasm thereby inducing ubiquitination and degradation of DIABLO [1,2]. DIABLO mRNA expression levels in neuroblastoma tumors are surprisingly high compared to other types of tumors and compared to normal tissue (Figure 3a). Moreover, we confirm an amplification of a region on the chromosome 12q arm in the neuroblastoma cell line NGP, which includes the DIABLO locus (Figure 3b) [42]. We therefore investigated whether the high BIRC6 expression allows neuroblastoma cells to survive the high levels of the pro-apoptotic protein DIABLO. DIABLO is a mitochondrial protein, which translocates to the cytoplasm after apoptotic stimuli where it can be degraded by BIRC6. We investigated the cellular localization of DIABLO and immunofluorescence indeed shows a clear localization of DIABLO within the mitochondria (Figure 3c), which is confirmed by cell fractionation showing that the majority of DIABLO is localized in the cell organelle fraction (Figure 3d). To test whether DIABLO can be functionally activated upon an apoptotic stimulus, we treated neuroblastoma cells with the BCL2 inhibitor ABT263, which results in stimulation of pore formation in the mitochondria [43]. Cell fractionation indicates that the cytosolic DIABLO levels increases 24 h after ABT263 treatment (Figure 3d), demonstrating that DIABLO can be released from the mitochondria and stimulate apoptosis.

Untreated cells have low cytoplasmic DIABLO levels (Figure 3d). These levels are probably restricted by BIRC6 activity. To test this hypothesis, we first investigated by a co-immunoprecipitation analysis whether BIRC6 and DIABLO physically interact in neuroblastoma cells. Cell lysates of SKNSH and IMR32 cells were immunoprecipitated with a BIRC6 antibody. Western blot analysis of these precipitates with a DIABLO antibody shows a strong signal at the correct position for DIABLO. This confirms a physical interaction between both proteins (Figure 3e). Finally, we investigated whether silencing of BIRC6 results in increased DIABLO protein levels. Western blot analysis of SKNSH cells treated with two different shRNAs for BIRC6 shows that the silencing of BIRC6 induced by both hairpins results in a clear increase of DIABLO protein levels (Figure 2f).

These experiments suggest that BIRC6 may effectively inactivate cytoplasmic DIABLO in neuroblastoma cells and can thereby prevent an apoptotic response.

## Discussion and conclusions

In this paper we analyzed aberrations in gene copy number and mRNA expression of genes directly involved in the intrinsic apoptotic pathway in neuroblastoma. BIRC6, known as an inhibitor of the pro-apoptotic protein DIABLO, showed gene copy number gains and increased expression. Silencing of BIRC6 with two shRNAs targeting different parts of the coding sequence resulted in increased cytoplasmic DIABLO levels and triggered apoptosis. As expected, BIRC6 directly interacted with DIABLO proteins.

The apoptotic response in neuroblastoma cells upon BIRC6 silencing occurs in a background of surprisingly high DIABLO expression levels. High cytoplasmic levels of DIABLO can be tumor inhibitory through binding of IAPs in the cytoplasm. We suggest two mechanisms why high DIABLO expression does not induce apoptosis in neuroblastoma. Firstly, we show that the major fraction of DIABLO protein has a mitochondrial localization. This mitochondrial sequestration is probably mediated by the exceptionally high levels of BCL2, which occurs in most neuroblastoma tumors [33]. Targeted inhibition of BCL2 caused an increase in cytoplasmic DIABLO levels, suggesting that sequestration of DIABLO in the mitochondria occurs by inhibition of pore formation through BCL2. Secondly, we showed that DIABLO is effectively bound by BIRC6 in neuroblastoma and that DIABLO levels increase upon silencing of BIRC6. This suggests an effective degradation of cytoplasmic DIABLO by BIRC6. We did not find a correlation between BIRC6 expression and prognosis or prognostic markers. We also did not find a correlation between BIRC6 and BCL2 or DIABLO mRNA expression (data not shown). This can be explained by the observation that the expression of both BCL2 and DIABLO is generally high in all neuroblastoma tumor samples and not in just a subset [33]. The function of high mitochondrial DIABLO levels in neuroblastoma remains elusive. Other pro-apoptotic proteins in the mitochondria, like cytochrome C, have shown to be involved in energy metabolism [44] but no such mechanism has been found for DIABLO yet.

The interaction of BIRC6 with CASP9, which has been reported previously [1,2,45], has not been validated in this paper. Since CASP9 has been shown to be functionally active in neuroblastoma despite its location on a frequently lost region [46], it would be interesting to investigate whether there is an additional inhibition of this protein by BIRC6 in neuroblastoma cells.

Release of the strongly increased mitochondrial DIABLO levels would offer a therapeutic potential in neuroblastoma. This suggests that the proteins that impair the pro-apoptotic function of DIABLO could be effective drug targets. The previously shown efficacy of targeted BCL2 inhibition could relate to high DIABLO levels [33]. Moreover, direct BIRC6 inhibition would also increase the cytoplasmic pro-apototic function of DIABLO. BIRC6 inhibitors are not available at this moment, but targeted drug development might be worth considering. BIRC6 is not located on the SRO of 2p, but it is frequently gained and functionally active and we therefore consider BIRC6 as a potentially important player in the dysfunction of apoptosis in neuroblastoma. The BIRC6 gene is validated as a novel therapeutic target. If BIRC6 inhibitors are developed, a combined inhibition of BIRC6 and BCL2 could yield synergistic effects.

## Competing interests

The authors declare that they have no competing interests.

## Authors’ contributions

All authors have made substantial contributions to conception and design, acquisition of data, analysis and interpretation of data. All authors have been involved in drafting the manuscript or revising it critically for important intellectual content and have given final approval of the version to be published.

## Pre-publication history

The pre-publication history for this paper can be accessed here:

http://www.biomedcentral.com/1471-2407/12/285/prepub
